# Left ventricular myocardial remodeling in dogs with mitral valve endocardiosis

**DOI:** 10.14202/vetworld.2020.731-738

**Published:** 2020-04-20

**Authors:** Yury A. Vatnikov, Andrey A. Rudenko, Boris V. Usha, Evgeny V. Kulikov, Elena A. Notina, Irina A. Bykova, Nadiya I. Khairova, Irina V. Bondareva, Victor N. Grishin, Andrey N. Zharov

**Affiliations:** 1 Department of Veterinary Medicine, Peoples’ Friendship University of Russia (RUDN University), 117198 Moscow, Russia; 2 Department of Veterinary Medicine, Moscow State University of Food Production, 125080 Moscow, Russia

**Keywords:** dogs, endocardiosis, heart failure, mitral regurgitation, myocardial remodeling

## Abstract

**Background and Aim:**

Left ventricular myocardial remodeling could play an important role in the progression of chronic heart failure (CHF) syndrome in dogs with mitral valve endocardiosis. The aim of this study was to evaluate the left ventricular myocardial remodeling in dogs with mitral valve endocardiosis and to study the dependence of the incidence of this pathological phenomenon on the functional class (FC) of progression of the CHF syndrome.

**Materials and Methods:**

A total of 108 afflicted dogs and 36 clinically healthy dogs were examined using transthoracic echocardiography. The following structural and geometric parameters of the left ventricular remodeling were evaluated: Myocardial mass and its index, sphericity index at the end of systole and diastole, end-systolic and end-diastolic relative wall thickness, and integral remodeling index.

**Results:**

In all clinically healthy dogs, a normal type of the left ventricular chamber geometry was revealed, whereas, in dogs with mitral valve endocardiosis, the normal geometry of the left ventricle occurred in 56.4%, eccentric hypertrophy in 24.1%, concentric remodeling in 10.2%, and concentric hypertrophy in 9.3% of the cases. In patients with endocardiosis, there was no dilatation type of cardiac remodeling observed.

**Conclusion:**

When compared to the clinically healthy animals, the dogs with mitral valve endocardiosis presented with indicators of structural and geometric remodeling, such as increased myocardial mass, myocardial mass index, and sphericity index at the end of systole and diastole, as well as relatively reduced integral systolic index of remodeling and systolic relative thickness of the walls of the heart. The parameters of the left ventricular myocardial remodeling correlated significantly with the FC of CHF syndrome.

## Introduction

Hereditary and acquired pathologies in animals of both non-infectious and contagious etiology are very diverse and complex, characterized by various forms of manifestations, which are often difficult to diagnose and require special approaches in treatment and prevention [1-6].

Endocardiosis of the mitral valve is caused by its myxomatous degeneration, and it is the most common among acquired heart diseases in dogs. The prevalence of this pathology is about 14-40% in the population of small breed dogs [3,7,8]. Endocardiosis in dogs is characterized by chronic myxomatous degeneration of the mitral valve, which leads to thickening and incomplete closure of its cusps and the development of mitral regurgitation whose severity is the main determinant of disease progression. It should be noted that most dogs with endocardiosis have an asymptomatic course for many years or even throughout their lives [4,9,10]. However, with progressive mitral regurgitation, severe complications can arise in the form of development of left-sided and then right-sided congestive chronic heart failure (CHF), usually secondary to the high pulmonary arterial hypertension syndrome [5]. However, in dogs with endocardiosis, the tricuspid valve is often affected leading to the development of tricuspid regurgitation [7,11]. The progressive course of the disease ultimately causes death of the animal or euthanasia due to severity of the symptoms, sharp deterioration in the quality of life, or development of cardiac decompensation that is refractory to therapy [3,12,13].

Based on the potential adverse outcomes and high prevalence of endocardiosis in dogs, its accurate and early diagnosis, and effective monitoring of the progression of CHF, it is a major problem in the veterinary medicine involving small domestic animals. Standard transthoracic echocardiography in dogs is currently regarded as a non-invasive diagnostic method for early detection of mitral valve lesions that assess the severity of mitral regurgitation as well as its effect on cardiac remodeling, left ventricular myocardial function, and indirect determination of pressure in the pulmonary artery [6,14,15].

The processes of the left ventricular remodeling in dogs with mitral valve endocardiosis begin in the preclinical stage and reach maximum changes in severe CHF [16-18]. Heart remodeling is a combination of molecular, cellular, and interstitial changes that are clinically manifested as changes in the size, shape, and function of the myocardium due to impaired cardiac activity [2,19,20]. In the scientific literature, there are two types of cardiac remodeling: Physiological (adaptive) remodeling and pathological remodeling [21,22]. This paper focuses on malignant pathological heart remodeling in dogs with mitral valve endocardiosis.

The clinical diagnosis of the left ventricular myocardial remodeling in animals and humans is based on identifying its morphological changes, especially the changes in cavity diameter, mass (hypertrophy and atrophy), and geometry (thickness and shape of the heart wall) using echocardiography or magnetic resonance imaging [2,23,24].

According to modern views on the neurohumoral mechanisms of the development of CHF, in addition to the activation of the sympathetic-adrenal system, the renin-angiotensin-aldosterone system, endothelial dysfunction, activation of inflammatory processes, oxidative stress, and endothelial dysfunction should be referred. These pathological processes stimulate the processes of remodeling, impairing hemodynamics, and retention of sodium and water in the bodies of humans and animals with CHF [18,22,25,26]. It is necessary to focus on the fact that the role of cardiac remodeling and its severity and effect on systolic function in the progression of CHF in humans and animals with mitral valve damage have not yet been established. It is also important to note that in the clinical veterinary medicine of small domestic animals, the features of the structural and functional remodeling of the left ventricular myocardium in mitral valve endocardiosis have not been studied enough.

Therefore, the study of patterns of the left ventricular remodeling in dogs with acquired valvular atrioventricular valve defects is relevant and can serve as a theoretical basis to develop effective methods that predict the effects of these pathologies and methods for early pathogenetic therapy.

The aim of this study was to evaluate the left ventricular myocardial remodeling in dogs with mitral valve endocardiosis and to study the dependence of the incidence of this pathological phenomenon on the functional class (FC) of progression of the CHF syndrome.

## Materials and Methods

### Ethical approval and informed consent

This study was performed in accordance with the Bioethical committee guidelines, and all the procedures were carried out pursuant to the Russian legislation on animal care and the European Guidelines on Animal Welfare. Owners’ informed consent were obtained for treatment and for the use of the data for research purposes.

### Animals and experimental design

This study was performed from 2017 to 2019 on the basis of the Veterinary Medicine departments of MGUPP and RUDN Universities in the Veterinary Clinic of the Institute of Veterinary Medicine, Veterinary Sanitary Expertise and Agro-Security of Moscow State University of Food Production (33 Talalikhina St., Moscow) and the Center of Veterinary Innovative Medicine (VIC) of the RUDN University (8k2 Miklukho-Maklaya Str., Moscow), private veterinary clinic “Avettura” (16k1, Kantemirovskaya Str., Moscow), private veterinary clinic “Epiona” (6k1/2 Marshal Zakharov Str., Moscow). It included dogs of small breeds afflicted with mitral valve endocardiosis (n=108), with body weight 4-19 kg (8.3±0.26) and aged 6-13 years (8.9±0.33). As a control group, clinically healthy dogs of small breeds (n=36), with body weight 3-17 kg (9.4±0.69) and age 7-12 years (8.7±0.85), were included in the study. Animals in groups were recruited as they arrived at the clinic.

The diagnosis of endocardiosis of the mitral valve in dogs was made comprehensively considering the data of anamnesis, clinical research methods, echocardiography, electrocardiography, and thoracic radiography [21]. The stages of endocardiosis in the sick dogs were determined according to the classification of the American College of Veterinary Internal Medicine (ACVIM). This classification was as follows: A (risk group), patients with no current evidence of heart disease but with a high risk of developing it in the future due to genetic disposition for endocardiosis (e.g., Pekingese, Chihuahua, Poodles, Terriers, and Taxies); B, asymptomatic stage; C, patients with symptoms of the disease; and D, patients refractory to standard therapy heart failure. It should be noted that Stage B is divided into two sub-stages: B1, endocardiosis without signs of remodeling of the left heart chambers (does not require therapy) and B2, disease accompanied by the left atrial expansion and eccentric left ventricular hypertrophy (preclinical stage of pathology requiring preventive therapy).

The FCs of CHF syndrome were evaluated based on the modified criterion of the New York Heart Association [12].

The criteria for the inclusion of dogs in the study included the presence of endocardiosis of the left atrioventricular valve with mitral regurgitation, complicated by CHF syndrome of different FCs.

The exclusion criteria were the presence of arterial hypertension (systolic blood pressure >180 mmHg), infective endocarditis, open arterial duct, dirofilariasis, cancer, hypothyroidism, chronic kidney disease, acute gastric dilatation, dilated cardiomyopathy, or incomplete clinic instrumental data.

Structural and geometric parameters of the left ventricle were assessed by performing an echocardiographic study in the B- and M-modes using standardized right and left parasternal positions along the long and short axes in the third to fifth intercostal space [27]. The study used the Mindray DP-50 ultrasound scanner (Shenzhen Mindray Bio-Medical Electronics Co. Ltd., China) with a microconvex ultrasonic multi-frequency sensor having a base scan rate of 5.0 MHz.

In the apical four-chamber view of the sectoral scan mode, the relative myocardial thickness of the left ventricle in systole (RMTs) and diastole (RMTd) was determined. The RMTs and RMTd were calculated as the ratio of the sum of the thickness of the free wall and interventricular septum at the end of systole and diastole, respectively, to the end-systolic or end-diastolic dimension of the left ventricle [7,19].

The end-systolic and end-diastolic dimensions of the left ventricle along the long and short axes were measured. The global sphericity indices (GSI) of the left ventricular chamber at the end of systole gonadosomatic indices (GSIs) and GSI diastole (GSId) were calculated as the ratio of the end-systolic and end-diastolic camera along the short and long axes, respectively [16,28].

The left ventricular mass (LVM) and the integral systolic remodeling index (ISRI) were calculated using Equations (3) and (7) [3,13]. The Left ventricular muscle mass index (LVMMI) was defined as the ratio of LVM to the surface area of the body [29]. The surface area of the body in dogs was determined by the calculation tables given in the publication “The Merck Veterinary Manual: 8^th^ edition” [30].


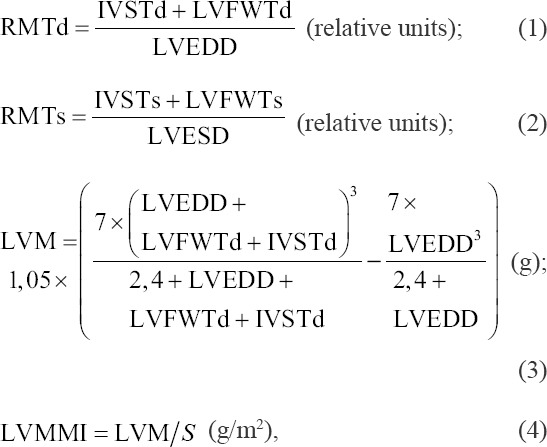



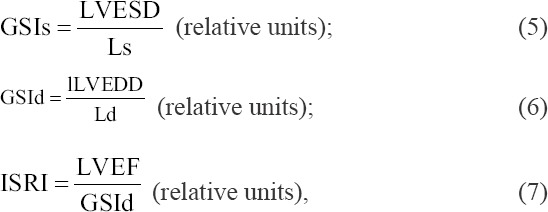


where S is the surface area of the body; LVFWT is the left ventricular free wall thickness in systole; LVFWTd is the left ventricular free wall thickness in diastole; IVSTs is the interventricular septum thickness in systole; IVSTd is the interventricular septum thickness in diastole; RMTd is the relative thickness of the walls of the left ventricle in diastole; RMTs is the relative thickness of the walls of the left ventricle in systole; LVESD is the left ventricular end-systolic dimension; LVEDD is the left ventricular end-diastolic dimension; LVEF is the left ventricular ejection fraction; Ls is the long axis of the left ventricle in systole; and Ld is the long axis of the left ventricle in diastole.

The criteria according to Ganau *et al*. [10] were used to determine the five types of structural and geometric remodeling of the left ventricular myocardium:

1. Normal geometry of the left ventricle (normal LVMMI and normal RMTd).
2. Dilatation type (normal LVMMI and reduced RMTd).
3. Concentric remodeling (normal LVMMI and increased RMTd).
4. Concentric hypertrophy (increase in LVMMI and RMTd).
5. Eccentric hypertrophy (increase in LVMMI with normal RMTd).

### Statistical analysis

The non-parametric Mann-Whitney U-test was used to compare the two groups, while the Kruskal–Wallis test was used to compare several groups. To compare the groups according to the frequency of occurrence of qualitative traits, the Chi-square criterion was used, and where necessary, the Yeats amendment was used. The relationship between the different FCs of CHF and left ventricular myocardial remodeling indices was established by the Spearman correlation method. The difference between groups of animals was considered significant if p<0.05. All calculations were performed on a personal computer using the statistical program STATISTICA 7.0 (StatSoft, USA).

## Results

The study included 144 dogs, of which 36 (25.0%), 37 (25.7%), 24 (16.7%), 36 (25.0%), and 11 (7.6%) animals were at Stages A, B1, B2, C, and D (ACVIM classification) of the pathology development, respectively.

Echocardiographic parameters characterizing the processes of remodeling of the myocardium of the left ventricle in dogs with mitral valve endocardiosis complicated by CHF syndrome of different FCs are listed in Table-1.

**Table-1:** Indicators of structural and functional remodeling of the left ventricular myocardium in dogs with endocardial mitral valve, depending on the functional class of chronic heart failure.

Marker	Clinically healthy dogs (n=36)	Sick dogs with the appropriate functional class of heart failure			

		I (n=38)	II (n=32)	III (n=27)	IV (n=11)
LVM, g	49.6±2.05	49.8±1.58	62.1±1.75*	65.2±1.77*	80.1±3.27*
LVMMI, g/m^2^	121.9±3.90	132.0±7.21	172.6±8.88*	162.2±7.50*	191.1±10.93*
GSIs, RU/ml	0.59±0.005	0.58±0.005	0.61±0.006*	0.65±0.011*	0.70±0.026*
GSId, RU/ml	0.53±0.005	0.53±0.005	0.58±0.009*	0.63±0.012*	0.71±0.020*
RMTd, RU/ml	0.65±0.015	0.64±0.015	0.68±0.011	0.64±0.019	0.64±0.018
RMTs, RU/ml	1.3±0.03	1.3±0.03	1.5±0.03*	1.2±0.04*	0.9±0.04*
ISRI, RU/ml	126.1±2.39	127.1±3.36	123.6±3.27	91.8±4.84*	53.9±4.71*

Numerator—M±m, Denominator—Lim;

* Significant difference compared with the control group (p<0.05). GSIs=Gonadosomatic indices, LVM=Left ventricular mass, LVMMI=Left ventricular mass index, ISRI=Integral systolic remodeling index

Changes in LVM in the endocardiosis afflicted dogs complicated by CHF of various FCs were found to be true as per the calculation of the Kruskal–Wallis analysis of variance (H=65.5; p<0.001). It was established that although the LVM during development of I FC of CHF syndrome did not differ from that of the control group, but it significantly increased (p<0.001) by 1.25, 1.31, and 1.62 times in the II, III, and IV FCs, respectively. In addition, LVM in sick dogs significantly correlated with the FCs of CHF syndrome (r=0.62; p<0.001).

Similarly, there were changes relative to LVMMI in endocardial dogs. The Kruskal–Wallis analysis revealed the presence of a significant difference in LVMMI between the animals of both groups (H=42.3; p<0.001). In dogs with endocardiosis, the LVMMI values significantly increased by 1.42 (U=202.5; p<0.001), 1.33 (U=162.0; p<0.001), and 1.57 (U=25.0; p<0.001) times with the development of CHF in the II, III, and IV FCs, respectively.

It is also necessary to add that the LVMMI reliably correlated with FCs of the CHF syndrome (r=0.51; p<0.001).

In patients with endocardiosis of the mitral valve, the GSIs and GSId significantly differed (p<0.001) in both animal groups (indicator H of the Kruskal–Wallis analysis was 51.2 and 69.8, respectively). With the development of III and IV FCs of the CHF syndrome in sick dogs, the indicators of GSIs and GSId increased significantly (p<0.001) when compared to clinically healthy dogs. It should be noted that these indices of the left ventricular myocardial remodeling significantly correlated with FCs of CHF (r<0.52; p<0.001).

In patients with endocardiosis, the RMTd was not significantly different in both groups. However, the RMTs value significantly changed in animals of the different FCs of CHF (H=55.7; r=0.64; p<0.001). In sick animals, RMTs ranged from 0.68 to 1.74 condition units with the development of II, III, and IV FCs of CHF showing averages of 1.5±0.03; 1.2±0.04, and 0.9±0.04 conditions units, respectively, and were significantly different (p<0.05) from that of clinically healthy dogs.

The ISRI index in dogs with mitral valve endocardiosis was found to be highly informative. Thus, the value of this parameter significantly decreased with the progression of CHF (H=42.0; p<0.01). Thus, in dogs with endocardiosis complicated by III and IV FCs of CHF, the value of this indicator was significantly reduced when compared to clinically healthy dogs. There was a negative correlation of ISRI in patients with FCs of CHF (r=−0.52; p<0.001). In the next stage, the type of the left ventricular remodeling in dogs with mitral valve endocardiosis using echocardiographic parameters of LVMMI and RMTd was noted (Table-2). Since the norms are not defined relative to these indicators for a sample of clinically healthy dogs of small breeds (n=36), we calculated reference values (±2*σ*) for LVMMI (76-168 g/m^2^) and RMTd (0.47-0.83 relative units).

**Table-2:** The frequency of detection of various types of the left ventricular remodeling in dogs with mitral valve endocardiosis, depending on the functional class of chronic heart failure.

Type of left ventricular myocardial remodeling	Clinically healthy dogs (n=36)	Sick dogs with the corresponding functional class of chronic heart failure			

		I (n=38)	II (n=32)	III (n=27)	IV (n=11)
Dilated cardiomyopathy	0/0	0/0	0/0	0/0	0/0
Concentric hypertrophy	0/0	1/2,6	5/15,6 *	3 /11,1	1/9,1
Eccentric hypertrophy	0/0	4 /10,5	7 /21,9*	8/29,6*	7/63,6*
Concentric Remodeling	0/0	7/18,4	2 /6,3	1/3,7	1/9,1
Normal heart	36/100,0	26/68,5*	18/56,2*	15/55,6*	2/18,2*

The numerator is abs. number; denominator -%;

* Significant difference compared with the control group (p≤0.05)

All clinically healthy dogs revealed a normal type of the left ventricular chamber geometry (Table-2). It should also be noted that none of the dogs included in this study revealed a dilatation type of left ventricular remodeling.

**Figure-1:**
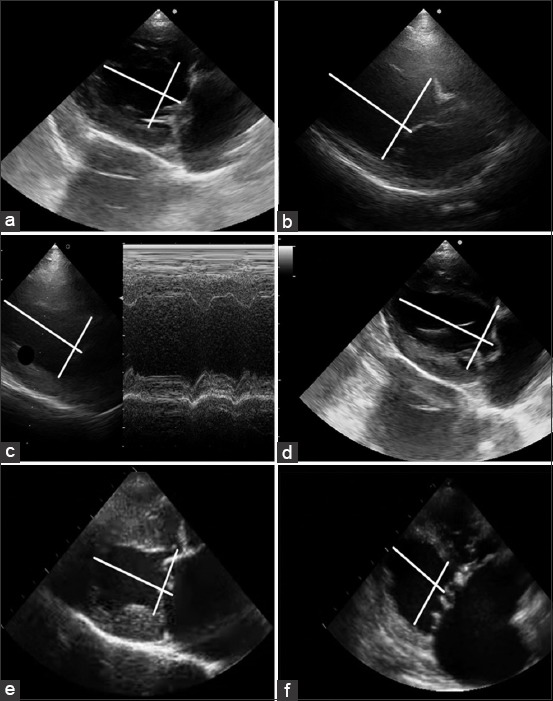
Left ventricular myocardial remodeling in dogs with mitral valve endocardiosis. (a, c) Normal geometry; (b, d, f) eccentric hypertrophy; and (e) concentric hypertrophy.

In dogs with mitral valve endocardiosis, the normal type of left ventricular geometry was significantly less common. The main type of left ventricular myocardial remodeling in such dogs was eccentric hypertrophy. Thus, these types of structural and geometric changes in the left ventricular myocardium in dogs with endocardiosis complicated by CHF syndrome of II, III, and IV FCs occurred with a frequency of 21.9 (Chi-square=6.57; p<0.05), 29.6 (Chi-square=9.69; p<0.05), and 63.6% (Chi-square=22.13; p<0.001), respectively. Concentric remodeling (10.2%) and concentric left ventricular myocardial hypertrophy (9.3%) were rarely observed in the dogs (Figure-1).

## Discussion

Mitral valve endocardiosis in dogs of small breeds is the most common nosological form of cardiovascular diseases [11,15]. This cardiac pathology in dogs has a genetic nature and may remain hidden for a long time, which could be attributed to the presence of a long pre-morbid period due to powerful compensatory mechanisms [11,31]. These physiological processes include activation of the neurohumoral systems, especially the sympathoadrenal system, renin-angiotensin-aldosterone system, endothelin, natriuretic peptides, and Frank-Starling mechanism [11,12,32]. Long-term activation of adaptive neurohumoral mechanisms leads to the depletion of the compensatory abilities of the body, development of decompensation, and progression of the CHF syndrome. Furthermore, hormones and biologically active substances exceeding the physiological norms of hemodynamic support act as toxins that damage the biological membranes of cells [33].

Symptoms of the left ventricular CHF in dogs with endocardiosis appear when blood stagnates in the pulmonary veins, and the disease starts with symptoms, such as shSortness of breath and decreased exercise tolerance (Stage C, ACVIM classification). Furthermore, significant dilatation of the left atrium can lead to compression of the left bronchus, and hence cough is often observed in sick dogs [34,35].

The concept of structural and geometric remodeling of the heart include any changes in its shape, structure, or size that arise against the background of myocardial damage of any origin or overload of the heart muscle, both by volume and blood pressure. In human clinical cardiology, the following types of structural and functional cardiac remodeling are distinguished: Dilatation, concentric hypertrophy, eccentric hypertrophy, concentric remodeling, and normal geometry [10].

Concentric remodeling is characterized by an increase in the wall thickness of the left ventricle and a decrease in its cavity without increasing the total mass of the myocardium. Concentric left ventricular hypertrophy is an increase in muscle mass and thickening of the left ventricular wall, which is formed when there is pressure overload on the heart muscle [7,29]. According to the results of our study, concentric remodeling (10.2%) and concentric left ventricular myocardial hypertrophy (9.3%) were rarely seen in sick dogs. Concentric remodeling indicates the adaptive nature of the process and the preservation of hemodynamics with a more favorable cylindrical shape of the left ventricle [23,36].

The eccentric type of remodeling is an increase in the left ventricular cavity and its mass due to volume myocardial overload. In endocardiosis of the mitral valve, the formation of this type of remodeling can be interpreted by retrograde systolic blood regurgitation into the cavity of the left atrium due to mitral insufficiency.

It should be noted that we have not identified cases of a dilated type of left ventricular remodeling in dogs with mitral valve endocardiosis, which corresponds to the data obtained by other authors [26,37].

In the early stages of endocardiosis of the mitral valve of the heart in dogs, adaptive myocardial remodeling develops that ensure the effective functioning of the Frank–Starling mechanism (an increase in cardiac output during a tonogenic expansion of the left ventricle). In the late stages of endocardiosis, the death of cardiomyocytes occurs, which leads to the development of maladaptive remodeling and a progressive course of the disease.

In human medicine, echocardiographic parameters LVMMI and RMTd can be used for determining the type of left ventricular remodeling [10]. In the scientific literature on clinical veterinary medicine, there is no information regarding the reference standards of these echocardiographic indicators of cardiac remodeling in dogs. Therefore, to determine the type of remodeling in dogs with mitral valve endocardiosis, we used the calculated reference values in relation to LVMMI and RMTd. Thus, our study used the following criteria to identify the type of the left ventricular remodeling in the afflicted dogs: Normal geometry of the left ventricle (LVMMI 76-168; RMTd 0.47-0.83 relative units); dilatation type (LVMMI 76-168; RMTd <0.47 relative units); concentric remodeling (LVMMI 76-168; RMTd >0.83 relative units); concentric hypertrophy (LVMMI >168; RMTd >0.83 relative units); and eccentric hypertrophy (LVMMI >168 g/m^2^; RMTd 0.47-0.83 relative units).

All clinically healthy dogs revealed a normal type of the left ventricular chamber geometry. In the endocardiosis dogs, the normal type of left ventricular geometry was significantly less frequent. The presence of the normal geometry of the left ventricle in sick dogs can be explained by the long preclinical course of endocardiosis, which corresponds to Stage B of the ACVIM classification.

Our study found that in dogs with mitral valve endocardiosis, the main type of remodeling was the eccentric hypertrophy of the left ventricular myocardium, which corresponds to the results of studies by other authors [9,14,38,39]. The indicators of structural and geometric remodeling (LVM, LVMMI, GSId, GSIs, and ISRI and RMTs) in sick dogs were significantly reduced as compared to healthy dogs. It should be noted that the indicated parameters of the left ventricular myocardial remodeling correlate significantly with the FCs of CHF. The evaluation of changes in RMTs has substantial practical importance. The increase in this parameter indicates the severity of diastolic dysfunction of the left ventricular myocardium, which does not depend on LVM. A decrease in RMTs may indicate an early decrease in cardiac output of the left ventricle, which is especially important in dogs with mitral valve endocardiosis [19,35,40].

In endocardiosis dogs, the ISRI index is highly informative. The progressive reduction of this parameter in sick dogs reflects the unfavorable association of the low contractile function of the left ventricle with a high degree of spherization.

The identification of types of left ventricular remodeling has a significant practical value since, in most cases, it determines the course, prognosis, and choice of tactics for treating dogs with mitral valve endocardiosis. A promising direction for further scientific research is the identification of a correlation between the types of the left ventricular myocardial remodeling and the types of its diastolic dysfunction in dogs with endocardiosis, depending on the FC of CHF syndrome.

## Conclusion

Thus, this research showed the processes of the left ventricular remodeling formation that was found in patients with mitral valve endocardiosis. The degree of myocardial remodeling in sick dogs correlates with the FC of CHF syndrome. When comparing patients with endocardiosis and clinically healthy individuals, the myocardial mass, myocardial mass index, and sphericity index at the end of systole and diastole are significantly increased, and the ISRI and the end-systolic relative wall thickness of the heart are significantly reduced in the former group. In clinically healthy dogs, only the normal type of the left ventricular chamber geometry was reported. In patients with endocardiosis, there was no dilatation type of cardiac remodeling, and the normal type of the left ventricular geometry (56.4%) and eccentric hypertrophy (24.1%) was common, whereas concentric remodeling (10.2%) and concentric hypertrophy (9.3%) were rarely observed.

## Authors’ Contributions

YuAV, AAR, and BVU had the original idea for the study and carried out the design. AAR, EVK, IVB, and NIK collected the samples. EAN, IAB, VNG, and ANZ analyzed the data. YuAV, BVU, and EVK drafted the manuscript. The final draft manuscript was revised by all authors. All authors read and approved the final manuscript.
